# Immunoproteasome modulates NLRP3 inflammasome‐mediated neuroinflammation under cerebral ischaemia and reperfusion conditions

**DOI:** 10.1111/jcmm.17104

**Published:** 2021-12-06

**Authors:** Xingyong Chen, Yinzhou Wang, Nannan Yao, Zejing Lin

**Affiliations:** ^1^ Department of Neurology Fujian Provincial Hospital Shengli Clinical Medical College of Fujian Medical University Fuzhou China; ^2^ Fujian Academy of Medical Science Fuzhou China

**Keywords:** immunoproteasome, ischaemic stroke, neuroinflammation, NLRP3 inflammasome, oxygen‐glucose deprivation

## Abstract

Compelling evidence showed that both nucleotide‐binding oligomerization domain‐like receptor family, pyrin domain‐containing protein 3 (NLRP3) inflammasomes and the immunoproteasome participate in neuroinflammatory responses in cerebral ischaemia injury. Moreover, inhibition of either NLRP3 inflammasomes or the immunoproteasome attenuates both neuroinflammation and neurological deterioration during ischaemic stroke. However, the underlying mechanism between the immunoproteasome and NLRP3 inflammasomes under ischaemic stroke conditions remains to be established. In this study, using both in vitro and in vivo ischaemic models, we demonstrated that the immunoproteasome inhibition reduced the expressions of NLRP3 inflammasome‐associated proteins, including NLRP3, apoptosis‐associated speck‐like protein (ASC), caspase‐1 and mature cytokines (interleukin [IL]‐1β and IL‐18). It also downregulated the levels of nuclear factor (NF)‐κB and pyroptotic‐ and apoptotic‐related proteins, and improved cell viability. In addition, inhibition of NF‐κB by the small molecule inhibitor Bay‐11‐7082 led to lower levels of NLRP3 inflammasomes and cleaved caspase‐1 proteins in BV2 cells after oxygen‐glucose deprivation and reoxygenation. Together, these findings suggest that the immunoproteasome may be responsible for inducing the expression and activation of NLRP3 inflammasomes via the NF‐κB pathway. Therapeutic interventions that target activation of the immunoproteasome/NF‐κB/NLRP3 inflammasome pathway may provide novel prospects for the future treatment of ischaemic stroke.

## 1 INTRODUCTION

Stroke is the second leading cause of death worldwide and a major cause of permanent disability. Inflammation and immune responses have emerged as important factors in both the onset and progression of stroke and can shape its clinical presentation and outcome.^1^ Recent research has revealed a new inflammatory mechanism that contributes to post‐stroke brain damage, which is mediated by nucleotide‐binding oligomerization domain‐like receptor family, pyrin domain‐containing protein 3 (NLRP3) inflammasomes. NLRP3 is a key component of pattern recognition receptors and plays a vital role in the inflammatory response by forming an intracellular multi‐protein complex known as the NLRP3 inflammasome.^2^ NLRP3 inflammasomes are activated in response to increased levels of NLRP3, apoptosis‐associated speck‐like protein (ASC), caspase‐1, and both interleukin (IL)‐1β and IL‐18 under both in vitro and in vivo ischaemic conditions. The inappropriate activation of NLRP3 can elicit a cascade of inflammatory responses and contributes to exacerbating neurovascular damage,^2^, ^3^, ^4^ while the inhibition of NLRP3 inflammasomes can improve the remodelling and integrity of the neurovascular unit.^5^, ^6^, ^7^ To date, the cellular localization of NLRP3 inflammasomes in the brain remains controversial.^3^ In addition, although there is evidence that nuclear factor (NF)‐kB and mitogen‐activated protein kinase (MAPK) signalling promotes neuronal NLRP inflammasome activation following ischaemic stroke,^8^ the precise mechanisms that contribute to post‐stroke NLRP3 inflammasomes activation remain unknown.

The ubiquitin‐proteasome system has a central role in the selective degradation of intracellular proteins that control inflammatory processes, cell cycle regulation and gene expression. Large amounts of data suggest that the ubiquitin‐proteasome system contributes to cerebral ischaemic injury, and that proteasome inhibition is a potential treatment for stroke. The proteasome complex consists of three different catalytically active subunits‐β1, β2 and β5 that are constitutively expressed. Elevated levels of interferons or oxidative stress induce the expression and incorporation of the catalytically active subunits β1i/low molecular mass polypeptide (LMP)2, β2i/LMP5 and β5i/LMP7 into newly formed immunoproteasome complexes. The catalytic β1i, β2i and β5i subunits display caspase‐, trypsin‐ and chymotrypsin‐like proteolytic activities, respectively.^9^ The immunoproteasome can have either a beneficial or a detrimental role depending on the experimental context. We have previously observed that the immunoproteasome participates in inflammatory mechanisms in ischaemic stroke, while inhibition of the immunoproteasome subunit LMP2 leads to lower NF‐κB expression in rat models of ischaemic stroke.^10^ ^,^ ^11^ Notably, a recent study reported that the toll‐like receptor 4 (TLR4)/NF‐κB/NLRP3 pathway plays an important role in sepsis‐induced myocardial dysfunction.^12^ We therefore hypothesized that immunoproteasome/NF‐κB/NLRP3 signalling might be involved in the inflammatory mechanisms of ischaemic stroke. In the present study, we aimed to investigate the expression of NLRP3 in the ischaemic brain and to confirm whether the immunoproteasome regulates the NLRP3 inflammasome pathway under conditions of cerebral ischaemia.

## 2 MATERIALS AND METHODS

### 2.1 Ethical approval and experimental animals

All experiments were approved by the ethics of Fujian Provincial Hospital and performed according to the guidelines of the US Department of Health for the Use and Care of Laboratory Animals. Male Sprague‐Dawley rats (weight 240–250 g) were randomly assigned into three groups (each group *n* = 10): sham operation group, LMP2‐shRNA group [rats underwent lentivirus‐mediated LMP2 short hairpin RNA (shRNA) injection], control‐shRNA group (rats underwent control lentivirus vector carrying scrambled shRNA injection).

### 2.2 MCAO/R model and lentiviral construction preparation and injection

Rats were anaesthetized and subjected to middle cerebral artery occlusion/reperfusion (MCAO/R) as described previously, with minor modifications,^10^ ^,^ ^13^ In brief, a midline neck incision was made, and the right common carotid artery, external carotid artery and internal carotid artery were isolated. The external carotid artery was tied. A 4–0 monofilament nylon suture (Beijing Sunbio Biotech Co. Ltd.) with a rounded tip was aseptically inserted from the right common carotid artery to the internal carotid artery through the stump of the external carotid artery and gently advanced to occlude the middle cerebral artery. Recirculation/reperfusion of the cerebral blood flow was allowed by gently removing the monofilament after 1 h of ischaemia, followed by 72 h of reperfusion. In sham‐operated animals, all procedures except occlusion of the MCA were performed. According to our previous study,^10^ the best‐performing LMP2‐shRNA sequence was CCTGGTCACCATTACAGCT, and the negative control scrambled shRNA sequence was CCATCATGGCTGTGGAATT (Genechem). A total volume of 10 μl of lentivirus suspension was delivered into the ipsilateral hemispheric region 3 days before MCAO using a 15 μl syringe at the following coordinates (10 μl/per site): bregma backward 1 mm, 1.5 mm lateral, 4 mm dorsoventral.^10^

### 2.3 Tissue preparation and TTC staining

At 72 h after reperfusion, five rats from each group were sacrificed after anaesthesia and then transcardially perfused with 0.9% sodium chloride at 4°C followed by 4% paraformaldehyde in 0.01 M phosphate‐buffered saline (PBS, pH 7.4). Brains were then removed, kept in the same fixative for 48 h at 4°C and cryoprotected in serial PBS isopropanol sucrose solutions (20% and 30%) at 4°C until brains sank. Coronal sections (10 μm) were cut on a cryostat (CM1900; Leica) and used for next experiments.

The slices were stained in 2% 2, 3, 5‐triphenyltetrazolium chloride (TTC) (Sigma‐Aldrich Corp) for 20 min at 37°C in the dark. Then, the infarcted brain tissue appeared white, whereas the noninfarcted region appeared red. The sections were fixed with 4% paraformaldehyde and then digitized.

### 2.4 BV2 cells culture and OGD/R

BV2 microglial cells were purchased from iCell Bioscience Inc. Complete media made up of Dulbecco’s Modified Eagle Medium (DMEM)/High Glucose containing 4 mM l‐glutamine and 4500 mg/L glucose (HyCloneTM; GE Healthcare Life Sciences), was supplied with 10% foetal bovine serum (FBS) (HyCloneTM; GE Healthcare Life Sciences) and 1% Penicillin/Streptomycin antibiotic (HyCloneTM; GE Healthcare Life Sciences) for maintenance of cell growth. The cell culture was maintained in a humidified incubator containing 5% CO_2_/95% air at 37°C. Experiments were conducted on cultures that reached about 80–90% confluence. To simulate ischaemic stroke conditions, BV2 cells were subjected to oxygen‐glucose deprivation/reoxygenation (OGD/R). Briefly, BV2 cells were washed with glucose‐free PBS twice and replaced with glucose‐free DMEM, and then were subjected to OGD for 3 h by placing cultures in oxygen‐free gas chambers equilibrated with 95% N_2_/5% CO_2_. The chamber was then sealed and kept at 37°C for the indicated time periods for hypoxia followed by a return to normoxic conditions with fresh normal medium (DMEM/F12 supplemented with 10% heat‐inactivated FBS, 100 U/ml penicillin and 100 mg/ml streptomycin, 5.5 mM glucose) for 24 h reoxygenation. Control cells incubated in DMEM/F12 solution were run in parallel for each condition for periods of time. The cells were harvested at time of point after reoxygenation.

### 2.5 BV2 cells pretreated with siRNA transfection

BV2 cells were seeded into six‐well plates at a density of 6 × 10^4^ cells/cm^2^ in 2 ml antibiotic‐free DMEM/F12 medium supplemented with FBS and then maintained at 37°C for 24 h until the cell confluence reached about 80%. For transfection, Lipofectamine™^2000^ (Invitrogen) was used according to the manufacturer’s instructions. The siRNA duplex targeting LMP2 (LMP2‐siRNA) was designed and synthesized by RiboBio Co., Ltd. as follows: target sequence: TGAAGAACATCTCCTACAA. The non‐silencing control sequence (scrambled control siRNA) was used as negative control (Control siRNA). The transfection cells were incubated for 6 h at 37°C in an environment with 5%CO_2_. Subsequently, the siRNA transfection medium was refreshed with antibiotic‐free DMEM/F12 medium and cells were incubated for another 24 h at 37°C in an environment with 5% CO_2_. Forty‐eight hours after transfection, cells were subjected to next experiments.

### 2.6 NF‐κB inhibitor Bay‐11‐7082 treatment of BV2 cells

NF‐κB inhibitor (Bay‐11‐7082) was purchased from Cayman Chemical. Briefly, cells were seeded in culture dishes and grown until 70% confluence. The medium was then replaced with a new medium containing either vehicle (0.1% DMSO) or Bay‐11‐7082 (10 μM) or in combination with LMP2‐siRNA transfection. Cells were exposed to hypoxia or left under normoxia for indicated time, and then were harvested at time of point for next experiments.

### 2.7 Assessment of cell viability by MTT

BV2 cells viability in response to OGD/R and different treatments was assessed by3‐(4, 5‐Dimethylthiazol‐2‐yl)‐2, 5‐diphenyl‐tetrazolium bromide (MTT) assays. Briefly, cells were seeded in 96‐well plates and were grown to about 80% confluence. 10 μl of the MTT reagent (0.5 mg/ml) was added into each well for 4 h to allow formation of purple formazan crystals. Absorbance (A) value was measured at an absorbance wavelength of 570 nm in a microplate reader within 30 min.

### 2.8 Immunofluorescence staining

Brain slides and cultured cells were detected with immunofluorescence staining as described previously.^11^ The following primary antibodies were used rabbit anti‐NLRP3, rabbit anti‐LMP2 and rat anti‐CD11b (1:400; Abcam), mouse anti‐NeuN (1:400; Chemicon), mouse anti‐glial fibrillary acid protein (GFAP) (1:500; Cell Signaling Technology) and mouse anti‐OX42 (1:300; Millipore). The different second antibodies were used as follows: anti‐rabbit IgG (H+L), F(ab’)2 Fragment (Alexa Fluor^®^ 488 conjugate) or anti‐mouse IgG (H+L), F(ab’)2 Fragment (Alexa Fluor^®^ 555 conjugate) or anti‐rabbit IgG (H+L), F(ab’)2 Fragment (Alexa Fluor^®^ 555 Conjugate) (1:1000; Cell Signaling Technology) or anti‐rat IgG‐H&L (Alexa Fluor^®^ 488) (1:1000; Abcam). If necessary, sections were counterstained for nuclei with 4′,6‐diamidino‐2‐phenylindole dihydrochloride (DAPI; 1:1000; Roche), and then slide mounted in ProLong^®^ Gold antifade reagent (Invitrogen) prior to imaging. In addition, propidium iodide (PI) is a laboratory reagent for the fluorescent staining of nucleic acids. Propidium iodide is useful for staining apoptotic cells and nuclei. PI (1:3000; Santa Cruz Biotechnology) was used in combination with fluorescent stain to observe cell viability via fluorescent microscopy. PI is excitable at 536 nm and Emits at 617 nm (red). The number of immunostaining‐positive cells was counted using Image‐Pro Plus image analysis software and presented as the average cell number per field on each section.

### 2.9 Terminal deoxynucleotidyl transferase‐mediated dUTP nick‐end labelling (TUNEL) assay

TUNEL staining was performed using an in situ cell death detection kit (Roche Applied Science) in accordance with the manufacturer’s instructions. Briefly, brain sections (10 μm thickness) were rinsed three times in PBS and then were incubated in 0.3% Triton X‐100 (v/v) in 0.01 M PBS (pH 7.4) for 20 min at room temperature. Subsequently, TUNEL reaction mixture was then applied for 60 min at 37°C. Fluorescence signal was detected using a fluorescence microscope at excitation/emission wavelengths of 492/520 nm (FITC, green).

### 2.10 Western blot analyses

Both ischaemic brain tissues and cells lysates were collected as previously described, and total protein (20–40 μg) was separated by 4–20% gradient SDS/PAGE (sodium dodecyl sulphate polyacrylamide gel electrophoresis) and then transferred onto polyvinylidene fluoride membranes (Millipore) using a Bio‐Rad Trans‐Blot^®^ transblot module (Bio‐Rad). The primary antibodies used were as follows: rabbit anti‐NLRP3, rabbit anti‐LMP2, rabbit anti‐procaspase‐1,p10,p12; rabbit anti‐IL‐18, rabbit anti‐IL‐1β, rabbit anti‐Gasdermin D(GSDMD), and rat anti‐CD11b (1:1000; Abcam), rabbit anti‐GSDMD (full length 53 kDa; cleaved part 35 kDa) (1:200, Affinity Biosciences), rabbit anti‐phospho‐NF‐κB p65 (Ser468) (1:1000; Cell Signaling Technology), rabbit anti‐caspase‐3 (This antibody is capable of recognizing the active form of caspase‐3 protein, molecular weight 17 kDa), mouse monoclonal anti‐ASC (1:300; Santa Cruz Biotechnology) and mouse monoclonal anti‐β‐actin (1:3000; Proteintech Group Inc.). The following secondary antibodies were used horseradish peroxidase (HRP)‐conjugated goat anti‐rabbit or HRP‐linked anti‐rat IgG (1:3000; Cell Signaling Technology), or HRP‐conjugated goat anti‐mouse IgG antibody (1:3000; Invitrogen). Immunoreactivity was detected with Chemiluminescent HRP Substrate (Millipore). The optical densities were normalized to that of β‐actin and calculated as target protein expression/β‐actin expression ratios (using software Image J v1.8.0).

### 2.11 Measurement of 20S proteasome activity and LDH

Proteasome activity assays were performed as previously reported,^10^ using a 20S Proteasome Activity Assay kit (APT280; Millipore) according to the protocol. In brief, 150 µg and 200 µg of protein (rats brains or cell lysates), respectively, were incubated in assay buffer including 250 mmol/L HEPES‐HCl, pH7.5, 5 mmol/L EDTA, 0.5% Nonidet‐P40 and 0.1% sodium dodecyl sulphate (SDS) (w/v), with 50 μmol/L different substrate in a final volume of 100 μl in 96‐well plates for 1 h at 37°C according to the manufacturer’s instructions.

The concentrations of lactate dehydrogenase (LDH) in the supernatants of each group of cells were examined in 96‐well plates using the Lactate Dehydrogenase Assay kit (Solarbio Life Science), according to the manufacturers’ instruction. The concentrations of LDH were measured for the absorbance at 450 nm using a microplate spectrophotometer and calculated according to the standard curve.

### 2.12 Statistical analysis

The data were expressed as means ± standard deviation (SD). Differences between groups were assessed by one‐way ANOVA analysis with a Tukey’s post hoc test. A value of *p* < 0.05 was considered statistically significant. IBM SPSS statistical software was used for statistical analysis (version 19.0; SPSS, Inc.).

## 3 RESULTS

### 3.1 Expression and cellular localization of NLRP3 inflammasomes in the brains of MCAO rats

**FIGURE 1:**
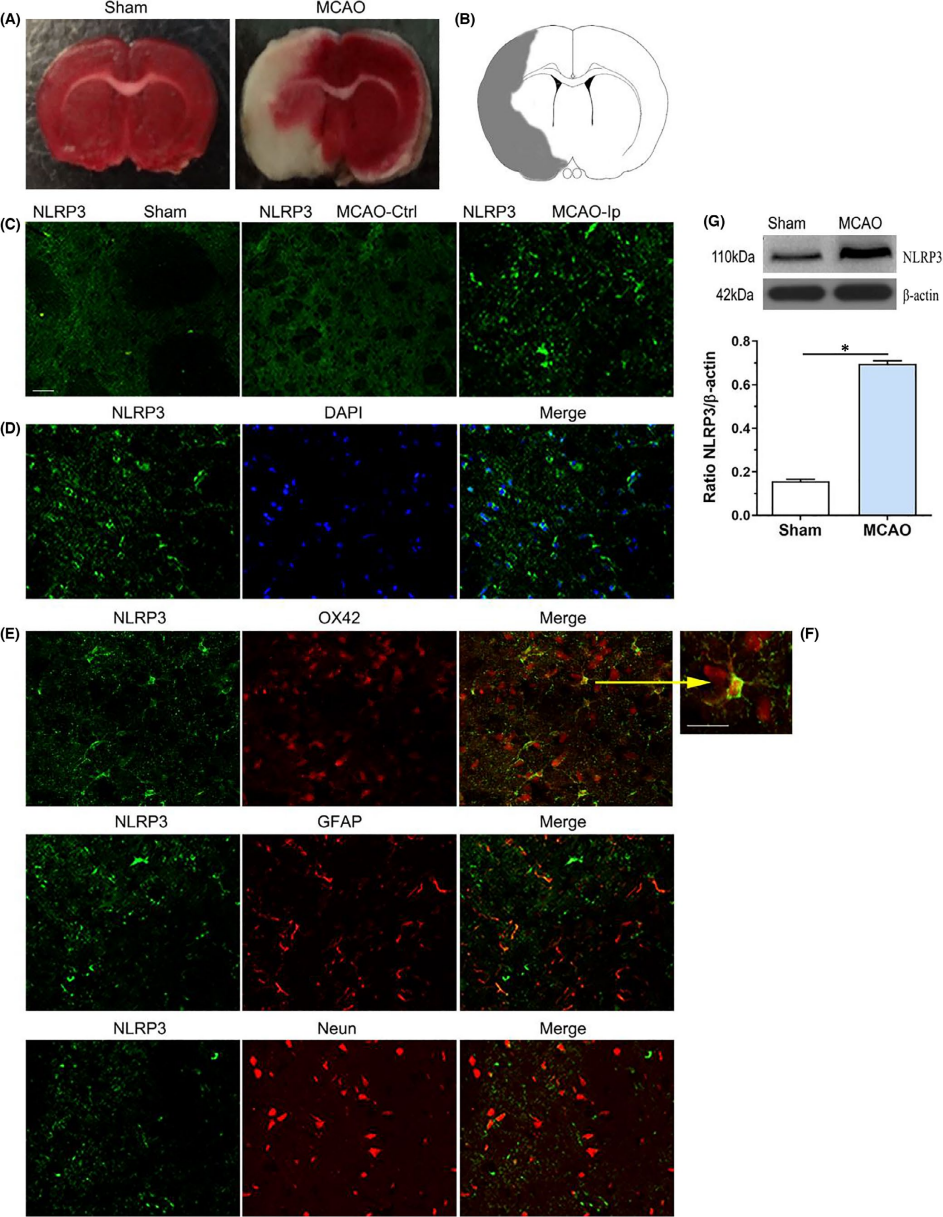
Expression and cellular localization of NLRP3 inflammasome in the brains of MCAO rats. (A) TTC staining showed the infarction brain tissue appeared white, whereas the noninfarcted region appeared red. (B) A schematic representation of a coronal brain section. The square fields represent observed regions. (C) The immunofluorescent showed the expression of NLRP3 in the sham and MCAO group. (D) NLRP3 was mostly present in the cytoplasm indicated co‐staining with DAPI. (E,F) Double immunostaining showed that the cell colocation of NLRP3 with OX42‐positive microglia/macrophage cells, glial fibrillary acid protein (GFAP)‐positive astrocytes and Neun‐positive neurons. (G) Quantification analysis of Western blotting showed the levels of NLRP3 inflammasome protein in different groups. *p < 0.001, compared with the sham group. Scale bars = 50 μm (images C–E). Scale bars = 100 μm (images F). sham: sham‐operated group; Ctrl: contralateral hemisphere, Ip: ipsilateral hemisphere. Data were presented as mean ± standard deviation (*n* = 5/group)

Because the expression and distribution of NLRP3 inflammasomes in the brain remain controversial, we first investigated NLRP3 inflammasome expression in the brains of rats after 1 h of MCAO and up to 72 h of reperfusion. After TTC staining, the infarction brain tissue appeared white, whereas the noninfarcted regions appeared red (Figure 1A). Using immunofluorescence, NLRP3 staining was barely detectable in the cortex and striatum of sham‐operated animals and in the contralateral hemisphere of ischaemic rats. In contrast, NLRP3 positivity was observed in the area surrounding the infarct core in the ipsilateral ischaemic hemisphere (Figure 1B,C). Co‐staining with 4′,6‐diamidino‐2‐phenylindole (DAPI) revealed that NLRP3 was mostly present in the cytoplasm (Figure 1D). To investigate the cell type(s) expressing NLRP3 inflammasomes, brain sections were then double‐labelled using antibodies against cell‐specific antigens. The majority of NLRP3‐positive staining occurred in OX42‐positive microglia/macrophage cells, although a few GFAP‐positive astrocytes and NeuN‐positive neurons also had NLRP3 staining (Figure 1E,F).

Western blotting confirmed that NLRP3 inflammasome protein level was significantly elevated after cerebral ischaemia/reperfusion. Quantification of the protein confirmed an approximate fourfold increased in NLRP3 inflammasome protein level in the MCAO group compared with the sham group (*p* < 0.001) (Figure 1G). Together, our findings indicate that ischaemia elevates NLRP3 inflammasome expression in brain cells, and especially in microglia.

### 3.2 Cerebral ischaemia induces the expression of proinflammatory cytokines and apoptotic‐ and pyroptotic‐related proteins

**FIGURE 2:**
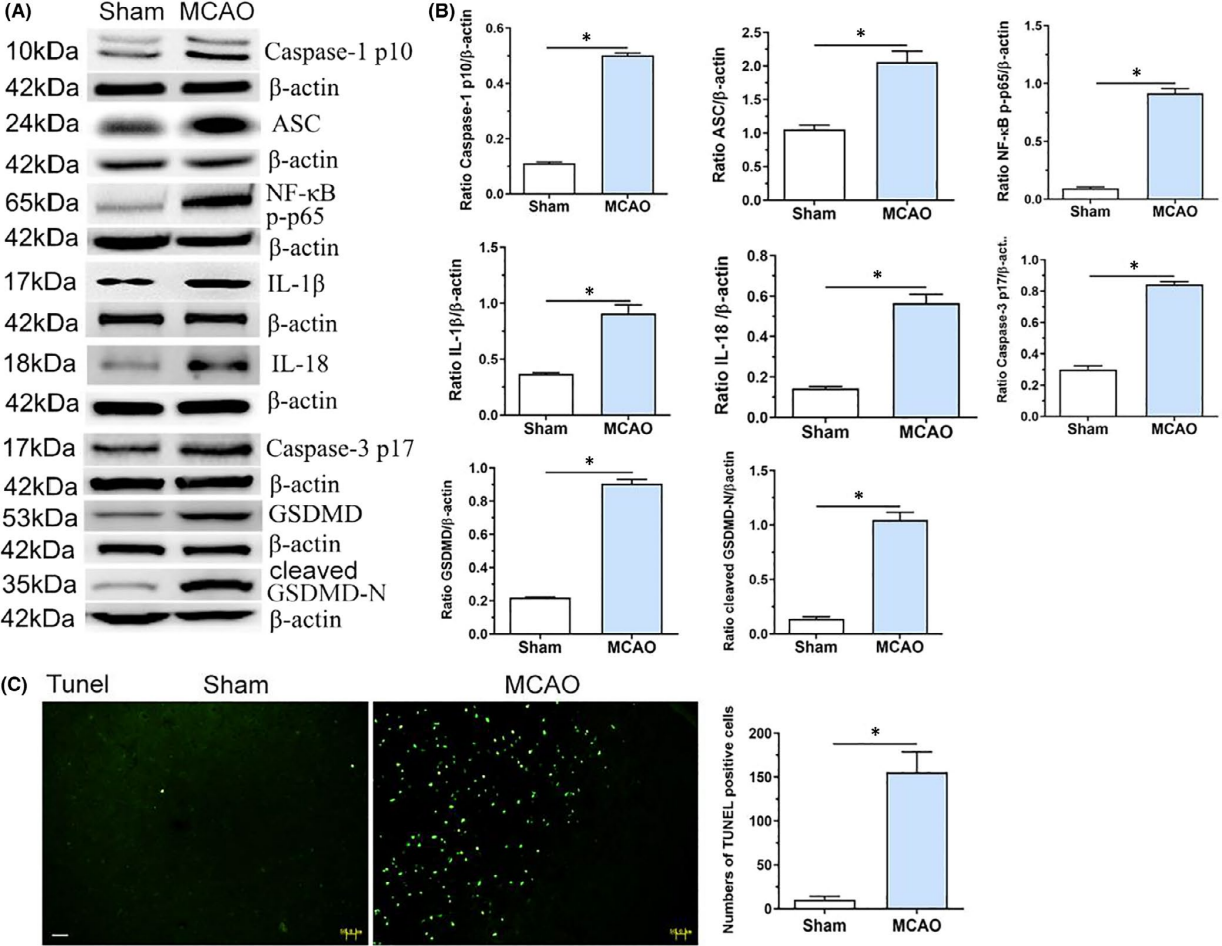
Cerebral ischaemia induces the expression of proinflammatory cytokines, apoptotic‐ and pyroptotic‐related proteins. (A, B) Western blotting indicated the expressions of cleave caspase‐1 p10, ASC, NF‐κB p‐p65, IL‐1β, IL‐18, cleaved caspase‐3 p17, full‐length and cleaved GSDMD‐N proteins in sham and MCAO group, respectively. (C) Tunel staining indicated the numbers of Tunel‐positive cells in the striatum area were predominantly increased in the MCAO group compared with the sham group.*p < 0.001, compared with the sham group. Scale bars = 50 μm. Sham: sham‐operated group; Data were presented as mean ± standard deviation (*n* = 5/group)

Cerebral ischaemia/reperfusion induced a significant elevation in the levels of inflammatory factors and apoptotic‐ and pyroptotic‐related proteins in the ipsilateral hemisphere from the ischaemia. Western blotting indicated that the protein levels of cleaved caspase‐1, ASC, phosphorylatedNF‐κB/p65 (NF‐κB p‐p65), and IL‐1β and IL‐18 were significantly upregulated in the MCAO group compared with the sham‐operated group. Furthermore, cerebral ischaemia/reperfusion induced the elevated expression of apoptotic‐ and pyroptotic‐related proteins. As expected, cleaved caspase‐3p17 (a marker of apoptosis) and gasdermin D (GSDMD; a marker of pyroptosis, including full‐length GSDMD and cleaved GSDMD‐N) proteins were also significantly upregulated in the MCAO group compared with the sham‐operated group (both *p* < 0.001) (Figure 2A,B). In addition, TUNEL staining revealed that the numbers of TUNEL‐positive cells were mainly higher in the MCAO group than in the sham group (*p* < 0.001) (Figure 2C). These data indicate that cerebral ischaemia induces NLRP3 activation and promotes cell death (including both apoptosis and pyroptosis).

### 3.3 Inhibiting the immunoproteasome subunit LMP2 downregulates the levels of inflammasome components, proinflammatory cytokines, and apoptotic‐ and pyroptotic‐related proteins

**FIGURE 3:**
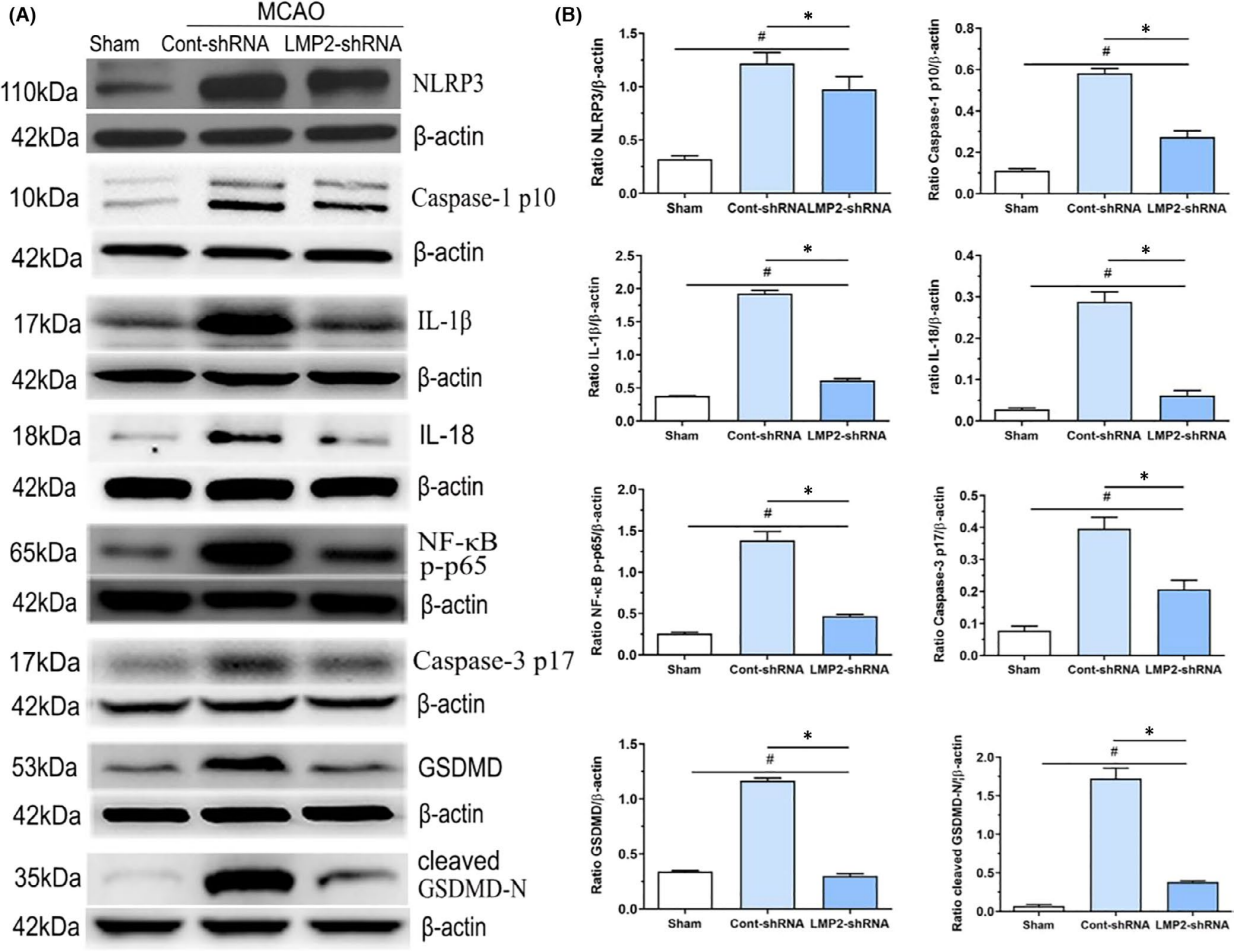
Inhibition of immunoproteasome LMP2 reduces the levels of inflammasome components, proinflammatory cytokines, apoptotic‐ and pyroptotic‐related proteins. (A,B) Western blotting indicated that NLRP3, cleave caspase‐1p10, NF‐κB p‐p65, IL‐1β, IL‐18, cleaved caspase‐3 p17, full‐length and cleaved GSDMD‐N protein levels in each group. ^#^ *p* < 0.001, compared with the sham group; ^*^ *p* < 0.001, compared with the control‐shRNA group. Sham: sham‐operated group; Cont‐shRNA: control‐shRNA group. Data were presented as mean ± standard deviation (*n* = 5/group)

In the present study, we confirmed that inhibiting the immunoproteasome subunit LMP2 using shRNA led to downregulated levels of both NLRP3 inflammasome components and proinflammatory cytokines. Western blotting demonstrated that the protein levels of NLRP3, cleaved caspase‐1p10, IL‐1β, IL‐18, NF‐κB p‐p65, cleaved caspase‐3 p17, total GSDMD, and cleaved GSDMD‐N were significantly lower in the LMP2‐shRNA group than in the control‐shRNA group (*p* < 0.001) (Figure 3A,B).

### 3.4 Effects of OGD/R on the morphology and viability of BV2 microglial cells

**FIGURE 4:**
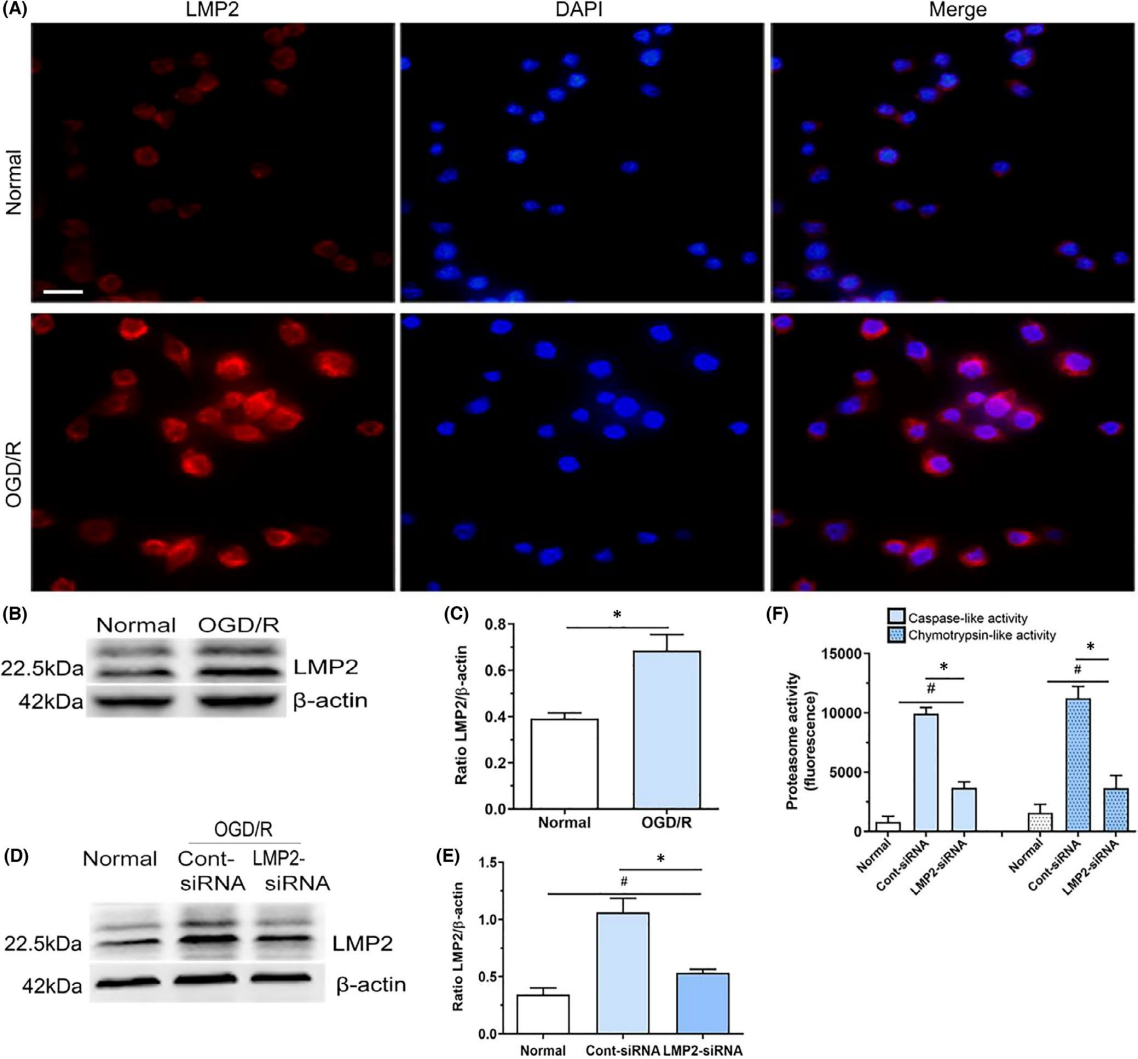
OGD/R significantly enhances the expression of immunoproteasome LMP2 and increases proteasome activities in BV2 cells, but was attenuated by LMP2‐siRNA transfection. (A) Immunofluorescent analysis showed the expression of LMP2 under OGD/R and normal culture conditions. (B,C) Western blotting indicated that LMP2 protein levels were significantly upregulated in the OGD/R group compared with the normal culture group. ^*^ *p* < 0.001, compared with the normal culture group. (D,E) Compared with control siRNA group, siRNA knockdown of LMP2 significantly reduced the levels of LMP2 protein in BV2 cells exposed to OGD/R. ^#^ *p* < 0.001, compared with the normal culture group; ^*^ *p* < 0.001, compared with control siRNA group. (F) The caspase‐like and chymotrypsin‐like proteolytic activities in BV2 cells under OGD/R and normal culture conditions. ^#^ *p* < 0.001, compared with the normal culture group; ^*^ *p* < 0.001, compared with control siRNA group. Scale bars = 50 μm. Results were expressed as mean ± standard deviation from six independent experiments. Cont siRNA, control siRNA; OGD/R, Oxygen‐glucose deprivation and reoxygenation

Oxygen‐glucose deprivation/reoxygenation‐treated BV2 microglial cells were enlarged and had many microspikes covering the surface of the cell body. They also displayed more intense immunoreactivity to the CD11b antigen compared with resting microglia, which had small, amoeboid shapes (provided as supplementary data Figure S1A,B). Western blotting showed that CD11b protein expression increased following OGD/R (*p* < 0.001) (Figure 4C, provided as supplementary data). Next, we investigated the effects of different durations of OGD on the viability of BV2 cells using MTT assay. After 1, 3, 6 and 9 h of OGD followed by 24 h of reoxygenation, cell viability decreased to approximately 87.8%, 67.3%, 44.6% and 32.6%, respectively (*p* < 0.001) (provided as supplementary data Figure S1D). We therefore selected OGD for 3 h as the optimal duration of OGD for the next investigation.

We next revealed that the LDH content was markedly increased after OGD/R, from 623.93 ± 52.74 U/L in BV2 cells under normal culture conditions to 1550.97 ± 90.83 U/L in cells under OGD/R conditions (*p* < 0.001) (Figure 4E, provided as supplementary data).

### 3.5 LMP2‐siRNA transfection reverses enhanced the immunoproteasome LMP2 expression and proteasome activity in BV2 cells following OGD/R

Compared with the normal culture group, immunoreactivity of the immunoproteasome subunit LMP2 was significantly enhanced in BV2 cells following OGD/R (Figure 4A). LMP2 was unevenly distributed throughout cells, with predominant localization in the cytosolic site of the nuclear envelope/endoplasmic reticulum membrane, as observed using co‐staining with DAPI (Figure 4A). Western blotting indicated that LMP2 protein level was significantly upregulated in the OGD/R group compared with the normal culture group (*p* < 0.001) (Figure 4B,C). In addition, compared with the scrambled siRNA group (control siRNA group), the knockdown of LMP2 by siRNA transfection significantly reduced the level of LMP2 protein in BV2 cells after exposure to OGD/R (Figure 4D,E) (*p* < 0.001).

**FIGURE 5:**
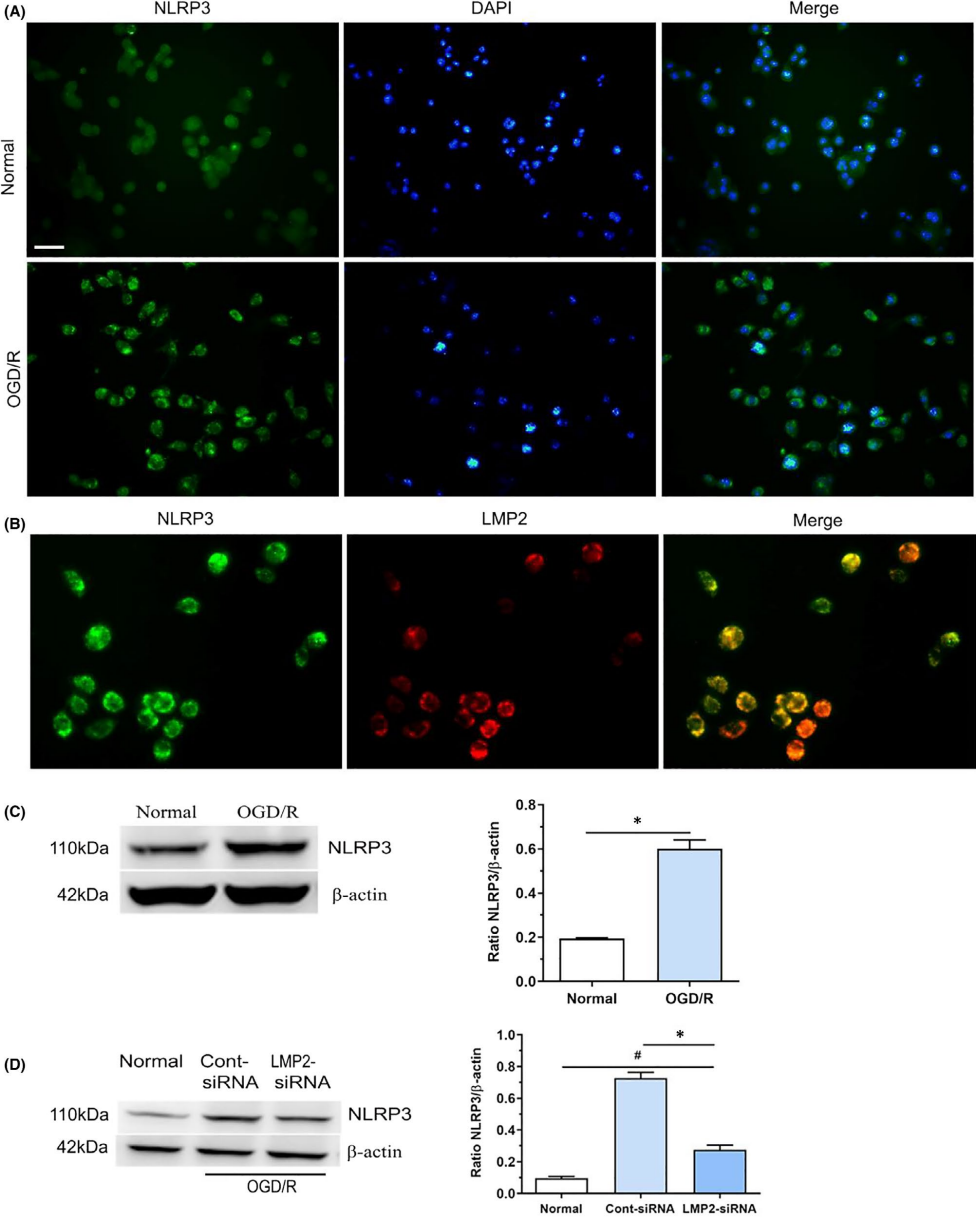
OGD/R induces expression of inflammasome NLRP3, but was attenuated by LMP2‐siRNA transfection. (A) Immunofluorescent showed OGD/R induces expression of NLRP3 inflammasome and with a predominant localization in the cytoplasm. (B) Mostly NLRP3‐positive cells co‐localized with LMP2‐positive cells. (C) Western blotting indicated that NLRP3 protein levels in the normal culture group and OGD/R group. *p < 0.001, compared with the normal culture group. (D) Western blotting showed that NLRP3 protein levels in the normal culture, LMP2‐siRNA and control siRNA group. ^#^ *p* < 0.001, compared with the normal culture group; ^*^ *p* < 0.001, compared with control siRNA group. Scale bars = 50 μm. Results were expressed as mean ± standard deviation from six independent experiments. Cont siRNA, control siRNA; OGD/R, Oxygen‐glucose deprivation and reoxygenation

The caspase‐like and chymotrypsin‐like activities of cell lysates at 24 h reoxygenation points under OGD/R were also measured. Compared with the normal culture group, the caspase‐like and chymotrypsin‐like proteolytic activities in BV2 cells significantly increased following OGD/R (Figure 5F). However, compared with the control siRNA group, pretreatment with LMP2‐siRNA reduced the proteolytic activity of the cell lysates, to approximately 37.1% and 32.4% in cells pretreated with LMP2‐siRNA and following OGD/R, respectively (*p* < 0.001) (Figure 4F). These results indicate that inhibiting LMP2 significantly reduces both the expression of LMP2 and the proteasome activity of BV2 cells under OGD/R conditions.

### 3.6 OGD/R induces NLRP3 inflammasome expression, but this effect is attenuated by LMP2‐siRNA transfection

Immunofluorescent revealed that OGD/R‐induced NLRP3 inflammasome expression with a predominant localization in the cytoplasm (Figure 5A). Notably, most NLRP3‐positive cells co‐localized with LMP2‐positive cells (Figure 5B). Furthermore, Western blotting showed that NLRP3 protein levels were significantly higher in the OGD/R group compared with the normal culture group (*p* < 0.001) (Figure 5C). However, compared with the control siRNA group, siRNA knockdown of LMP2 significantly downregulated NLRP3 protein levels in BV2 cells following OGD/R (*p* < 0.001) (Figure 5D). These results indicate that inhibiting the immunoproteasome LMP2 significantly reduces NLRP3 expression under OGD/R conditions.

### 3.7 OGD/R induces the expression of caspase‐1, ASC, NF‐κB, IL‐1β, IL‐18, caspase‐3 and GSDMD in BV2 cells, but this effect is attenuated by LMP2‐siRNA transfection

**FIGURE 6:**
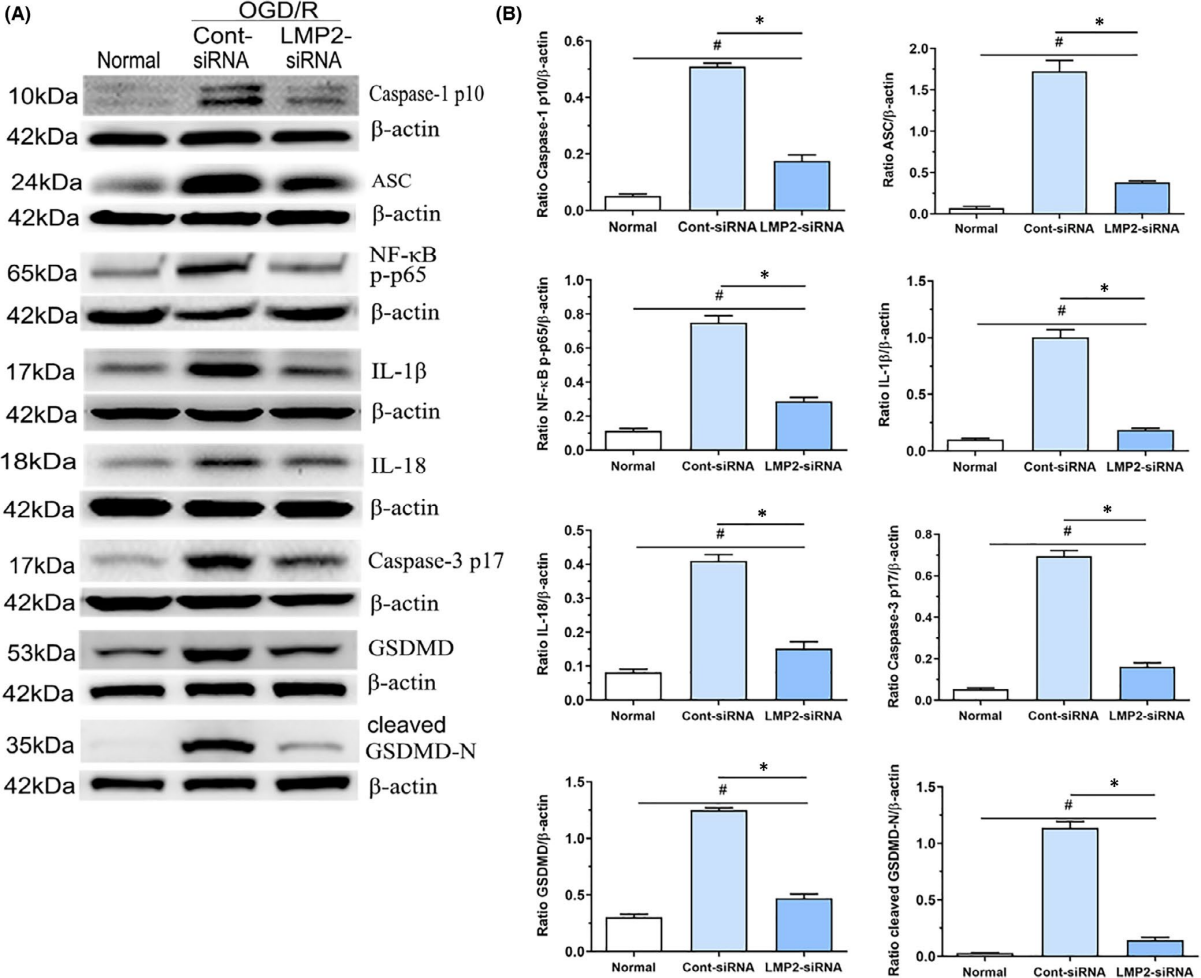
OGD/R induces the expression of caspase‐1, ASC, NF‐κB, IL‐1β, IL‐18, caspase‐3 and GSDMD in BV2 cells, but was attenuated by genetic knockdown of LMP2. (A,B) Western blotting showed that caspase‐1 p10, ASC, NF‐κB p‐p65, IL‐1β, IL‐18, cleaved caspase‐3 p17, full‐length GSDMD and cleaved GSDMD‐N protein levels in BV2 in the normal culture, LMP2‐siRNA and control siRNA group, respectively. ^#^ *p* < 0.001, compared with the normal culture group; ^*^ *p* < 0.001, compared with control siRNA group. Results were expressed as mean ± standard deviation from six independent experiments. Cont siRNA, control siRNA; OGD/R, Oxygen‐glucose deprivation and reoxygenation

Western blotting demonstrated that, in BV2 cells, the protein levels of caspase‐1 p10, ASC, NF‐κB p‐p65, IL‐1β, IL‐18, cleaved caspase‐3 p17, GSDMD and cleaved GSDMD‐N were significantly upregulated in the OGD/R group compared with the normal culture group (*p* < 0.001) (Figure 6A,B). However, compared with the control siRNA group, siRNA knockdown of LMP2 significantly reduced the levels of these proteins in BV2 cells exposed to OGD/R for 24 h (*p* < 0.001) (Figure 6A,B).

### 3.8 Effects of immunoproteasome inhibition on the viability of BV2 cells following OGD/R

**FIGURE 7:**
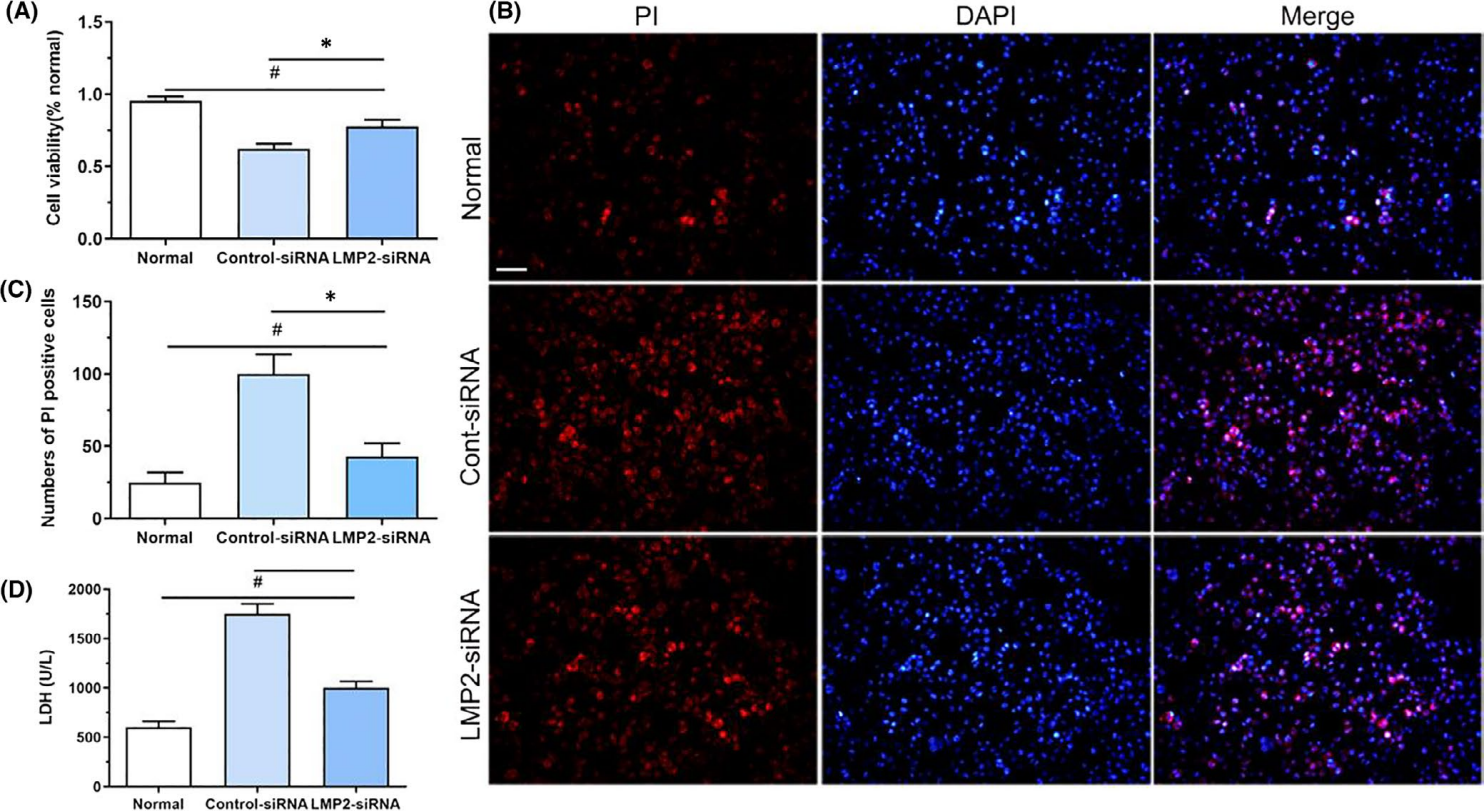
Inhibition of immunoproteasome LMP2 improves the viability of BV2 under OGD/R. (A) MTT assay showed cell survival under different conditions. ^#^ *p* *< *0.001, compared with normal culture group; ^*^ *p* < 0.001, compared with control siRNA group. (B,C) OGD/R induced more PI‐positive cells compared with normal culture group. Compared with the control siRNA group, the numbers of PI‐positive cells significantly decreased in LMP2‐siRNA group. (D) The levels of LDH were measured in the normal culture, LMP2‐siRNA and control siRNA group. ^#^ *p* < 0.001, compared with normal culture group; ^*^ *p* < 0.001, compared with control siRNA group. Scale bars = 50 μm. Results were expressed as mean ± standard deviation from six independent experiments. Cont siRNA, control siRNA; OGD/R, Oxygen‐glucose deprivation and reoxygenation

The MTT assay demonstrated that the use of LMP2‐siRNA to inhibit the immunoproteasome subunit LMP2 led to better BV2 cell survival under OGD/R. The MTT absorbance value was significantly higher in the LMP2‐siRNA group compared with the control siRNA group (*p* < 0.001) (Figure 7A). Furthermore, PI staining consistently indicated that OGD/R induced more PI‐positive cells compared with the normal culture group; however, inhibition of the immunoproteasome LMP2 improved the survival of BV2 cells under OGD/R. There were fewer PI‐positive cells in the LMP2‐siRNA group than in the control siRNA group (*p* < 0.001) (Figure 7B,C).

Compared with the normal culture group (LDH content 602.74 ± 60.51 U/L), the LDH content was significantly increased under OGD/R conditions, while the LDH content was significantly lower in the LMP2‐siRNA group (1003.14 ± 63.06 U/L) than in the control siRNA group (1750.64 ± 99.76 U/L) (*p* < 0.001) (Figure 7D). These data suggest that inhibition of the immunoproteasome LMP2 significantly improves cell survival under OGD/R conditions.

### 3.9 Inhibition of NF‐κB attenuates NLRP3 inflammasome expression following OGD/R

Next, we investigated whether inhibiting the NF‐κB pathway in vitro using the pharmacological inhibitor Bay‐11‐7082 leads to the attenuation of NLRP3 inflammasome expression, as was observed in BV2 microglial cells following OGD/R. Treatment with Bay‐11‐7082 reduced the protein levels of phosphorylated NF‐κB p65, NLRP3 and caspase‐1 compared with the vehicle control treatment (*p* < 0.001) (provided as supplementary data Figure S2A–D). In addition, pretreatment with Bay‐11‐7082 combined with LMP2‐siRNA significantly downregulated the levels of phosphorylated NF‐κB p65, NLRP3 and caspase‐1 proteins in BV2 cells exposed to OGD/R (*p* < 0.001) (provided as supplementary data Figure S2E,H).

## 4 DISCUSSION

In the present study, ischaemia‐induced NLRP3 inflammasome expression in brain cells, especially microglia, under both in vitro and in vivo ischaemic models. Furthermore, inhibition of the immunoproteasome subunit LMP2 significantly reduced the expression of NLRP3 inflammasome‐associated proteins, including NLRP3, ASC, caspase‐1, inflammation cytokines (IL‐1β and IL‐18), NF‐κB and pyroptotic‐ and apoptosis‐related proteins, and improved cell viability. In addition, the inhibition of NF‐κB with the pharmacological inhibitor Bay‐11‐7082 contributed to the attenuation of NLRP3 inflammasome and caspase‐1 protein expression in BV2 microglial cells after OGD/R. These data suggest that the immunoproteasome modulates the expression and activation of NLRP3 inflammasome proteins possibly through the NF‐κB signalling pathway.

The inflammasome is a vital part of the innate immune response to stroke. NLRP3 is the most intensively studied inflammasome and has been investigated in the cerebral ischaemia‐ or haemorrhage‐induced inflammatory response.^14^ ^,^ ^15^ To date, the expression and distribution of NLRP3 inflammasomes in the brain remain controversial. Both our and other studies have indicated that NLRP3 is mainly expressed in microglia and is hardly detected in neurons and astrocytes. This indicates that microglia might be a major producer of NLRP3 inflammasomes.^16^ In contrast, other studies have reported NLRP3 labelling in neurons.^17^ ^,^ ^18^ Thus, the cell localization of NLRP3 inflammasome remains uncertain, and the reason for the differences in localization in different studies is still unclear. Different models of ischaemic stroke, different durations of ischaemic insults and different interventions may all be possible explanations.

The NLRP3 inflammasome may have a key role in detecting cellular damage and mediating inflammatory responses after stroke.^2^ ^,^ ^19^ Activation of the inflammasome is usually necessary; the innate immune response to an insult efficiently protects against disease and death. However, excessive activation of the NLRP3 inflammasome can elicit a cascade of inflammatory responses, eventually leading to cell death (pyroptosis). Conversely, knockout of the *NLRP3* gene or pharmacological inhibition of the NLRP3 inflammasome using MCC950 or intravenous immunoglobulin G can attenuate the expression of NLRP3 inflammasome proteins and can also downregulate IL‐1β, IL‐18 and proapoptotic protein cleaved caspase‐3, thus largely avoiding neuronal deterioration and preserving blood‐brain barrier permeability and cerebral function in ischaemic stroke models.^5^, ^6^, ^7^ In the current study, we provided new evidence to support the idea that NLRP3 inflammasomes were upregulated in response to elevated levels of cleaved caspase‐1 and caspase‐3, ASC, GSDMD, IL‐1β and IL‐18 under in vitro and in vivo ischaemic conditions. Moreover, inhibition of the immunoproteasome subunit LMP2 led to significantly reduced levels of these proteins and improved cell viability following focal cerebral ischaemia or OGD/R.

So far, the exact molecular mechanisms underlying the protective effects of NLRP3 inflammasome inhibition against cerebral ischaemia/reperfusion injury remain largely unclear. A range of complex factors and mechanisms involve in the assembly and activation of the NLRP3 inflammasome, including the NF‐κB and MAPK signalling pathways,^8^ ^,^ ^20^ In the present study, we also explored that the pharmacological inhibition of NF‐κB decreased the NLRP3 inflammasome levels. These findings suggest that NLRP3 inflammasome expression may lie downstream of the NF‐κB signalling pathway. That is, the NF‐κB signalling pathway may regulate the production or activation of NLRP3 inflammasomes. Interestingly, in this study, although we found that immunoproteasome inhibition via LMP2‐shRNA to some extent decreased the expression of NLRP3; results were more convincing when LMP2‐shRNA and NF‐κB inhibitor combined. So it seems only immunoproteasome inhibition may not be enough to suppress neuroinflammation via the NLRP3 inflammasome pathway. In addition, some studies have provided strong experimental evidence supporting a role for immunoproteasome in modulating NF‐κB signalling under conditions of ischaemic stroke or inflammation response,^10^ ^,^ ^21^ Immunoproteasome‐mediated proteolysis has emerged as an important molecular mechanism in the regulation of wide‐ranging functions, both immune (such as antigen presentation) and non‐immune (including antioxidative stress),^9^ ^,^ ^22^ Thus, we revealed a mechanism by which the immunoproteasome regulate the expression of NLRP3 inflammasomes by facilitating the NF‐κB signalling pathway activation.

The present study has several limitations. Although we previously observed that inhibition of immunoproteasome LMP2 by shRNA reduced infarction volumes and improved the neurologic recovery in rats after 14 days ischaemia/reperfusion,^11^ the neurological deficit results of these genetic and pharmacological manipulations after stroke did not provide in this study. Theoretically, neurobehavioral changes can more directly and effectively reflect the effects of these interventions. In addition, the immunoproteasome has three specific catalytic subunits LMP2, LMP5 and LMP7 with apparently diverse functions. Previous studies have reported that LMP7 expresses in microglia and plays an important role of inflammatory in some disease models.^23^ ^,^ ^24^ Undoubtedly, further studies are required to fully understand the diverse roles and mechanisms by which the immunoproteasome inhibition against cerebral ischaemic injury.

In summary, the present study provides evidence that ischaemia induces the expression of NLRP3 inflammasomes, especially in microglia, and contributes to the inflammatory response and cell death. The immunoproteasome may regulate the expression of NLRP3 inflammasomes via the NF‐κB signalling pathway. Together, these findings suggest that therapeutic interventions that target the immunoproteasome/NF‐κB/NLRP3 inflammasome pathway may provide new opportunities for the future treatment of ischaemic stroke.

## CONFLICT OF INTEREST

The authors confirm that there are no conflicts of interest.

## AUTHOR CONTRIBUTIONS

**Xingyong Chen:** Conceptualization (equal); data curation (equal); formal analysis (equal); funding acquisition (equal); resources (equal); supervision (equal); validation (equal); writing – original draft (equal); writing – review and editing (equal). **Yinzhou Wang:** Conceptualization (equal); funding acquisition (equal); resources (equal). **Nannan Yao:** Investigation (equal); methodology (equal); resources (equal). **Zejing Lin:** Investigation (equal); methodology (equal); resources (equal).

## Supporting information

Fig S1‐S2

## Data Availability

The data that support the findings of this study are available from the corresponding author upon reasonable request.
