# Supplementary material for: Immunoproteasome modulates NLRP3 inflammasome‐mediated neuroinflammation under cerebral ischaemia and reperfusion conditions
Source: J Cell Mol Med. 2021 Dec 6;26(2):462–74. doi: 10.1111/jcmm.17104 (PMC8743645; doi:10.1111/jcmm.17104)
Supplement: Supplementary file 1 — Fig S1‐S2 [file JCMM-26-462-s001.docx]

**Supplementary data**

**Supplementary Fgure1 Effects of OGD/R on BV2 microglia cells morphological changes and viability**

**
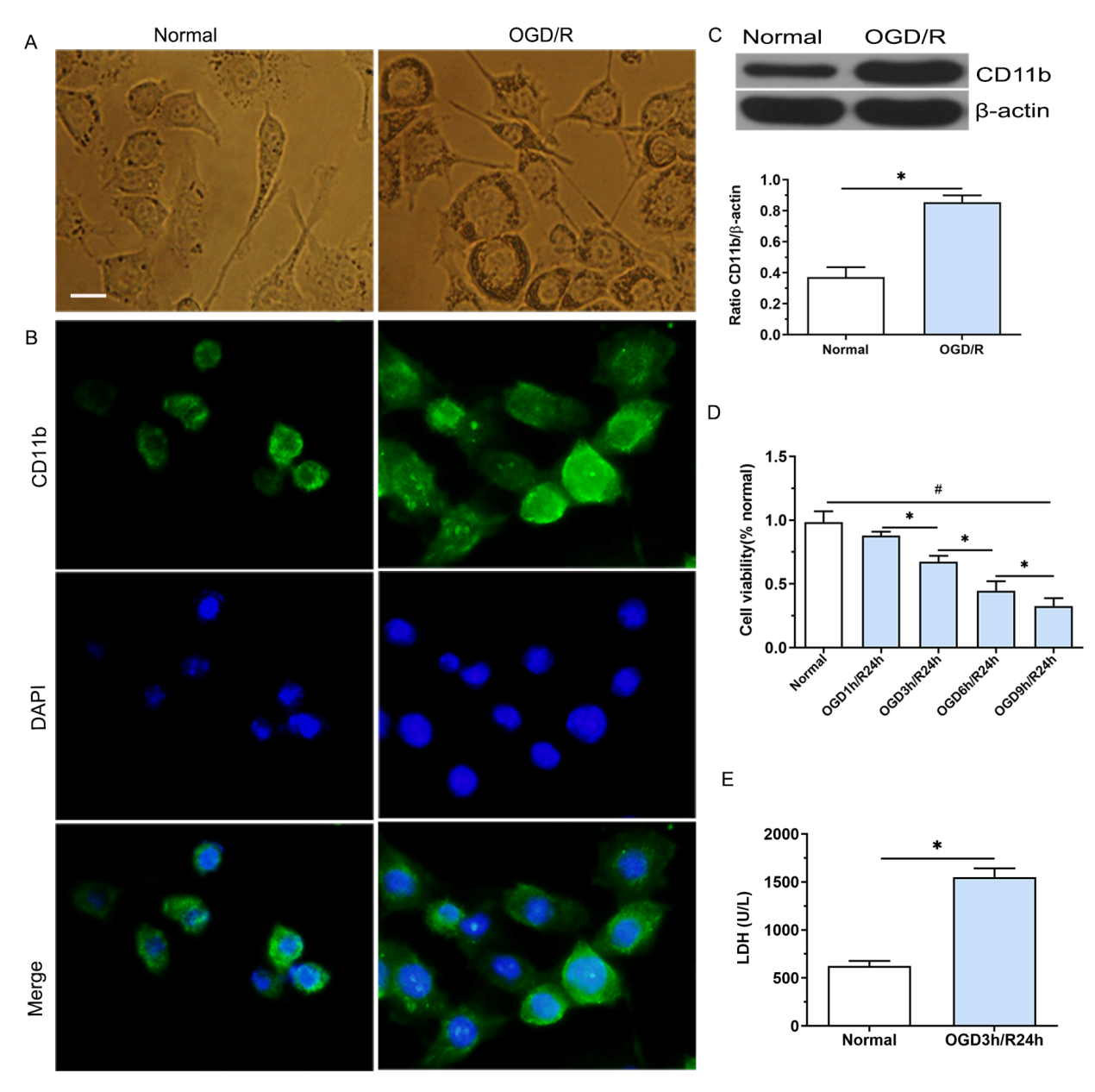
**

(A~B)OGD/R-induced BV2 microglia cells were enlarger and have many microspikes covering cell body surfaces, and displayed more intense immunoreactivity due to CD11b antigen, as compared to resting microglia with small, amoeboid shapes. (C)Western blotting showed enhanced expression of CD11b protein following OGD/R. ^*^P<0.001, compared with the normal culture group. (D) The effects of different time duration of OGD on BV2 cells viability with MTT assay. ^#^P<0.001, compared with the normal culture group; ^*^P<0.001, compared with the previous time group. (E) The concentration of LDH was markedly increased in cells under OGD for 3 hours followed by 24 hours reoxygenation. ^*^P<0.001, compared with the normal culture group. Scale bars = 50 μm. Results were expressed as mean ± standard deviation from six independent experiments. OGD/R: Oxygen-glucose deprivation and reoxygenation.

**Supplementary Figure2 Inhibition of NF-κB attenuates NLRP3 inflammasome expression following OGD/R**

**
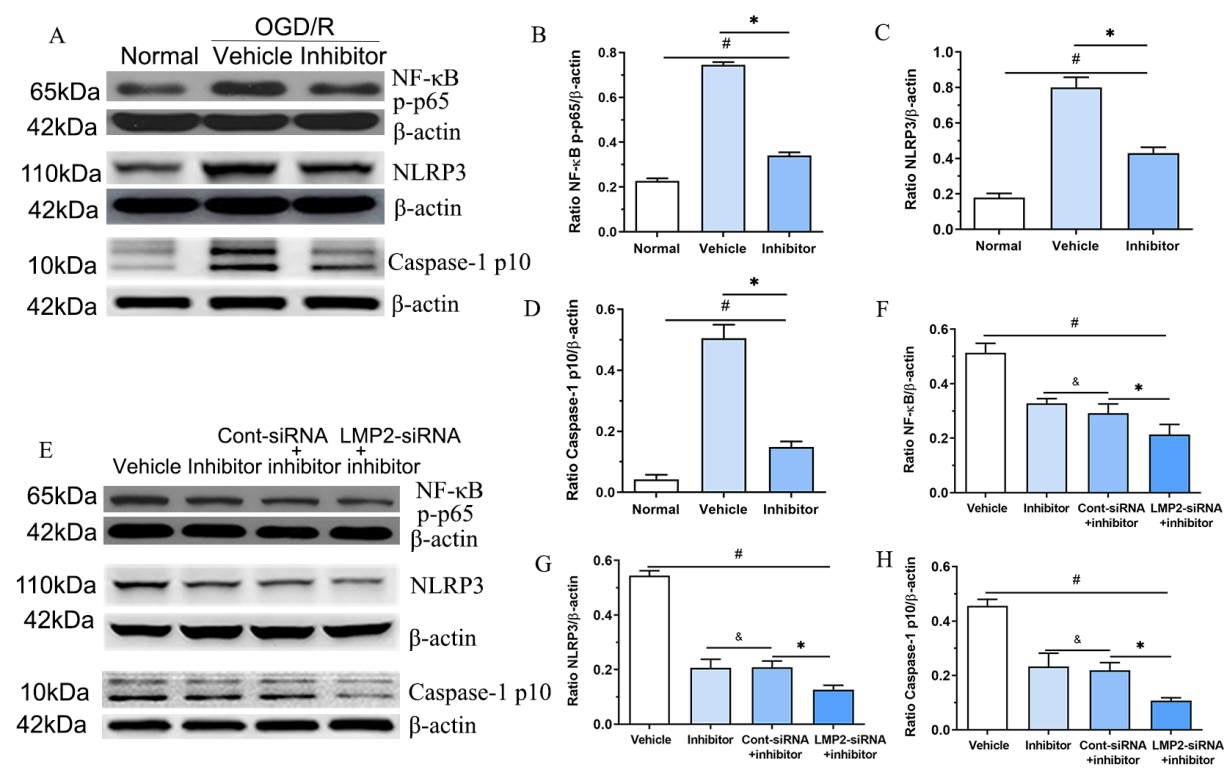
**

(A~D)Inhibitor Bay-11-7082 reduced levels of phosphorylated NF-κB-p65, NLRP3 and caspase-1 compared to the vehicle controls. ^#^P<0.001, compared with normal culture group; ^*^P<0.001, compared with control-siRNA group. (E~H) Pretreatment with Bay-11-7082 combined with LMP2-siRNA significantly down-regulated levels of phosphorylated NF-κB-p65, NLRP3 and caspase-1 proteins in BV2 cells exposed to OGD/R, respectively. ^#^P<0.001, compared with the vehicle group; ^&^P>0.05, compared with the inhibitor group; *P<0.001, comparison between inhibitor+cont-siRNA group with inhibitor+LMP2-siRNA group). Results were expressed as mean ± standard deviation from six independent experiments. Cont-siRNA: control siRNA. OGD/R: Oxygen-glucose deprivation and reoxygenation.
